# Resolution of Crizotinib-Associated Fulminant Hepatitis following Cessation of Treatment

**DOI:** 10.1155/2018/3413592

**Published:** 2018-08-05

**Authors:** Gregory W. Charville, Sukhmani K. Padda, Richard K. Sibley, Ajithkumar Puthillath, Paul Y. Kwo

**Affiliations:** ^1^Department of Pathology, Stanford University School of Medicine, Stanford, CA 94305, USA; ^2^Department of Medicine, Division of Oncology, Stanford University School of Medicine, Stanford, CA 94305, USA; ^3^Stockton Hematology Oncology Medical Group, Stockton, CA 95204, USA; ^4^Department of Medicine, Division of Gastroenterology and Hepatology, Stanford University School of Medicine, Stanford, CA 94305, USA

## Abstract

Targeted cancer treatments offer the prospect of precise inhibition of tumor growth without the untoward off-target toxicity of traditional chemotherapies. Still, unintended, often idiosyncratic side effects, such as drug-induced liver injury, can occur. We discuss the case of a 26-year-old female with a history of* ROS1*-rearranged lung adenocarcinoma, undergoing treatment with the tyrosine kinase inhibitor crizotinib, who presented to our hospital with abdominal pain and scleral icterus. Liver chemistries were notable for hyperbilirubinemia (5 mg/dL total) and marked transaminasemia (AST 1736 U/L, ALT >3500 U/L); liver biopsy demonstrated acute hepatitis with extensive necrosis. There was no evidence of an infectious or autoimmune etiology. It was discovered that the patient was taking a 500 mg once daily dose of crizotinib, in lieu of the intended dose of 250 mg twice daily. After immediate cessation of crizotinib therapy upon hospital admission, there was complete biochemical resolution of the hepatitis. This case highlights the potential reversibility of fulminant crizotinib-associated hepatoxicity, possibly related to supratherapeutic dosing, when managed with abrupt stoppage of the drug and initiation of supportive care.

## 1. Introduction

Crizotinib is a small-molecule inhibitor of the protooncogene receptor tyrosine kinases anaplastic lymphoma kinase (ALK), ROS1, and MET. As a targeted therapy, crizotinib has received approval for treatment of a distinct subgroup of non-small-cell lung cancers mediated by rearrangements of* ALK* or* ROS1 *[1, 2]. Most patients with* ALK*- or* ROS1*-rearranged lung cancers show an objective response to tyrosine kinase inhibition, which now represents a first-line approach to the treatment of these unique tumors [3]. The most common side effects of crizotinib include nausea, diarrhea, visual disturbances, fatigue, anorexia, constipation, abdominal pain, and upper respiratory tract infection, among others [3]. Elevated aminotransferases of varying degree have been reported as a side effect in trials of crizotinib. A much less common, and sometimes irreversible, side effect of crizotinib is fulminant hepatitis.

## 2. Case Report

A 26-year-old female presented to our hospital with a primary complaint of intermittent, progressive right upper quadrant abdominal pain of one week's duration, which coincided with the onset of darkened urine and yellowing of the eyes. There was mild nausea, but no emesis. The patient had no subjective fever or rashes. She perceived no significant swelling or unusual bleeding. She had no chest pain or myalgia. Her appetite was normal.

The past medical history was notable for a diagnosis of primary lung adenocarcinoma by bronchoscopic biopsy of a right lower lobe radiographic consolidation four months prior to this presentation. By immunohistochemistry, the adenocarcinoma expressed cytokeratin 7, thyroid transcription factor-1 (TTF-1), and napsin-A, but did not express cytokeratin 20, GATA-3, or PAX8, findings consistent with adenocarcinoma of lung origin. Fluorescence in situ hybridization (FISH) analysis of the primary tumor showed cytogenetic evidence of a* ROS1* gene rearrangement. No actionable mutations were detected by exon-targeted sequencing. Staging positron emission tomography-computed tomography studies revealed a fludeoxyglucose- (FDG-) avid right lower lobe lung mass with additional consolidation/nodularity in the right middle lobe, left upper lobe, and lingula, and no evidence of mediastinal or hilar lymphadenopathy; however, endobronchial ultrasound-guided biopsy of a level ten lymph node showed involvement by metastatic disease. MRI of the brain showed no evidence of intracranial metastasis.

Given the presence of a* ROS1* gene rearrangement, the patient was started on oral crizotinib therapy (250 mg twice daily) 10 weeks prior to her presentation. One week after the initiation of crizotinib therapy, the patient was admitted to another hospital with chest pain, subjective fever, and emesis. She was diagnosed with a bacterial pneumonia and discharged from the hospital with instructions to complete seven-day courses of levofloxacin and metronidazole and a ten-day course of fluconazole, while decreasing her crizotinib dose to 250 mg once daily. One week after discharge from this preceding hospitalization, the patient's liver chemistries were normal: total bilirubin 0.2 mg/dL, AST (SGOT) 15 U/L, ALT (SGPT) 25 U/L, and alkaline phosphatase 58 U/L. Five weeks and two days prior to the current presentation, after the resolution of the abovementioned symptoms, the patient was instructed to increase the crizotinib dose back to the original 250 mg twice per day. However, on admission to our hospital, the patient reported restarting crizotinib at one dose of 500 mg daily, as she found the side effects of gastrointestinal upset more tolerable with this self-imposed regimen. The patient denied other new medications, toxin exposures, or drug/alcohol use. There were no known sick contacts or significant travel.

On physical examination, the vital signs were normal with blood pressure 107/69, heart rate 75 beats per minute, temperature 36.8°C (oral), respiratory rate 12 per minute, and oxygen saturation 96% on room air. The patient was not in distress. The eyes showed mild scleral icterus and the oropharynx was without lesions. Both the rate and rhythm of the heart were regular; there were no heart murmurs. The lungs were bilaterally clear to auscultation: there were no wheezes or rales. The patient exhibited mild epigastric and right upper quadrant abdominal pain on examination, but there was no palpable mass or organomegaly. Examination of the extremities showed no cyanosis or edema. The skin itself was not jaundiced and did not display spider angiomata or palmar erythema. There was no asterixis.

Liver chemistries at the time of admission showed total bilirubin 4.5 mg/dL (3.5 mg/dL conjugated, 1.5 mg/dL unconjugated), AST 1736 U/L, ALT >3500 U/L, and alkaline phosphatase 144 U/L. The INR was above the threshold of normal at 2.2. The white blood cell count was 10.6 K/*μ*L. The platelet count was 195 K/*μ*L. PCR studies of the serum for hepatitis B virus, hepatitis C virus, herpes simplex viruses- (HSV-) 1 and 2, and Epstein-Barr virus (EBV) were negative. Serologic studies for anti-hepatitis A virus antibody (IgM), anti-hepatitis C virus antibody (IgG), anti-hepatitis B virus core antibody (IgM), and hepatitis B surface antigen were all negative. Anti-varicella zoster virus IgG antibody was present, while IgM antibody was not. Analysis of the serum for anti-nuclear antibody was negative (titer <1:80), as were studies for anti-liver-kidney-microsomal antibody and anti-smooth muscle antibody. Serum acetaminophen levels were below the limit of detection (<2.0 *μ*g/mL). Ceruloplasmin was normal at 27.7 mg/dL.

Computed tomography imaging with the aid of intravenous contrast was notable for periportal edema with a small amount of perihepatic fluid and gallbladder mural edema. There were no intra- or extrahepatic mass lesions. Significant interval decrease in the radiographic burden of disease in the right lung was noted with minimal ongoing ground-glass opacities in the right upper and right middle lobes.

**Figure 1:**
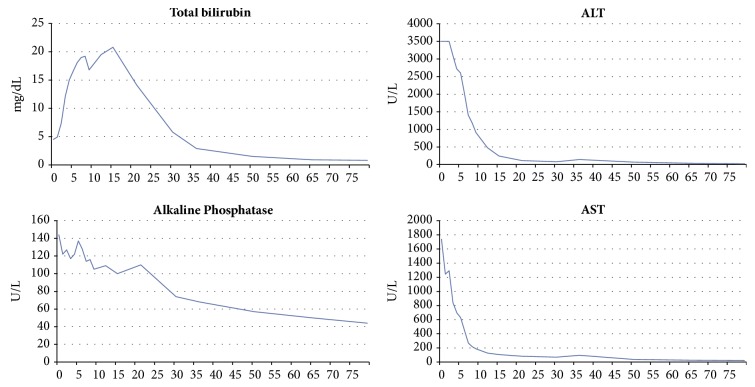
Time-course of liver function studies. The x-axis spans from Day 0 (day of admission and cessation of crizotinib therapy) to Day 79.

Given suspicion of crizotinib-associated hepatotoxicity, targeted therapy was discontinued on admission to our hospital. A continuous infusion of N-acetylcysteine was started (6.25 mg/kg/hr). The AST showed immediate improvement, peaking on the first day of admission (Figure 1). The ALT remained at the maximum limit of detection until the third day of admission and then precipitously declined. Six days after admission, results of serologic studies showed mildly elevated anti-HSV IgM (1.35; normal <1.10); anti-HSV-1 IgG was also present, while anti-HSV-2 IgG was not. With this information, intravenous acyclovir (10 mg/kg every eight hours) was initiated.

**Figure 2:**
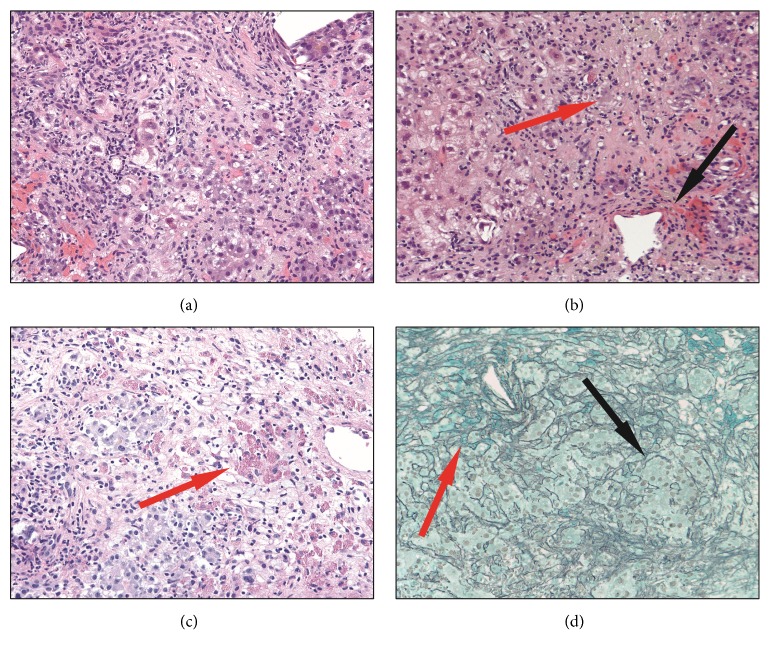
(a) H&E-stained histologic section of the liver biopsy demonstrating severe acute hepatitis characterized by a panlobular mixed inflammatory infiltrate with extensive hepatocyte necrosis (200x magnification). (b) H&E-stained histologic section of the liver biopsy demonstrating bridging parenchymal necrosis (red arrow) and cholestasis (black arrow) (200x magnification). (c) Periodic acid-Schiff stained histologic section of the liver biopsy with diastase pretreatment highlighting numerous ceroid pigment-laden macrophages (red arrow) (200x magnification). (d) Reticulin-stained histologic section of the liver biopsy demonstrating areas of parenchymal collapse (red arrow) and regenerative thickening of the hepatocyte plates (black arrow) (100x magnification).

On the seventh day of hospitalization, a transjugular liver biopsy was performed. Histologic sections of the liver biopsy were notable for a predominantly lymphohistiocytic panlobular inflammatory infiltrate accompanied by swaths of parenchymal dropout (Figures 2(a) and 2(b)). The inflammatory infiltrate included abundant pigment-laden macrophages and the occasional plasma cell. Granulocytes, including the rare eosinophil, were also seen. The sampled portal tracts showed the appropriate constellation of artery, vein, and interlobular bile duct without evidence of ductopenia. The inflammation was not centered on the portal tracts and the bile ducts themselves showed no obvious intraepithelial infiltrate or reactive-type epithelial changes. Numerous degenerating and necrotic hepatocytes were seen. Those hepatocytes that remained showed reactive cytoplasmic vacuolization and mitotic activity suggestive of a regenerative response. Periodic acid-Schiff (PAS) stain with diastase pretreatment drew attention to the ceroid pigment-laden Kupffer cells indicative of the significant hepatocyte death that must have preceded this biopsy (Figure 2(c)). The PAS-positive intracytoplasmic aggregates of alpha-1-antitrypsin deficiency were not seen. Cholestasis was also prominent. Reticulin and trichrome stains highlighted areas of hepatocyte loss and parenchymal collapse (Figure 2(d)). There was no significant fibrosis by trichrome stain. Hepatic stores of copper and iron were not significantly increased on special stains for each element. The periportal copper deposition of chronic cholestasis was not present. No viral cytopathic effects were identified; immunohistochemical stains for HSV and adenovirus were both negative. Furthermore, analysis of biopsied tissue by PCR showed no evidence of varicella zoster virus, HSV-1/2, cytomegalovirus, or EBV. Nonspecific viral culture of the liver biopsy also showed no viral cytopathic effect.

Acyclovir was discontinued eight days after admission (two days following its initiation), at which time the total bilirubin also peaked. N-acetylcysteine was discontinued and the patient was discharged to home nine days after admission with continually downtrending liver chemistries. She developed edema and ascites post discharge and required furosemide 40 mg daily and spironolactone 100 mg daily for three weeks until these symptoms resolved. Despite this, the liver tests continued to normalize during the patient's subsequent outpatient treatment. She never developed hepatic encephalopathy. All liver chemistries had normalized at follow-up 79 days after admission (Figure 1). Computed tomography studies at that time showed an interval increase in the right lower lobe ground-glass opacity, along with small nodules throughout the remainder of the bilateral lungs; the radiographic appearance of the liver was improved. Given evidence of residual cancer burden, a chemotherapeutic regimen of carboplatin and pemetrexed was initiated.

## 3. Discussion

Liver injury of varying degree is a documented side effect of crizotinib anticancer therapy. In 171 patients receiving first-line crizotinib, Solomon* et al*. observed elevated aminotransferases in 61 (36%), with 14% of patients showing Grades 3-4 transaminasemia [3]. Of these 24 patients with Grades 3-4 aminotransferase increases, all but four were successfully managed with dose interruptions or reductions. Those four outstanding cases required complete cessation of therapy. In a phase 1 study of crizotinib therapy in 79 pediatric patients (aged 1-22 years) with various malignancies, ALT elevations were seen in 62% and AST elevations in 56% [4]. Although none of the 899 patients with “definite, highly likely, or probable” drug-induced liver injury in the Drug-Induced Liver Injury Network's prospective study were attributed to crizotinib, these studies largely preceded the widespread implementation of the drug [5].

In addition to these more commonplace and modest aminotransferase abnormalities, individual cases of fulminant hepatic failure in the setting of crizotinib therapy have been reported. Sato* et al*. describe the case of a 54-year-old female presenting with fulminant hepatitis 29 days following the initiation of crizotinib therapy [6]. The patient died seven days after presentation despite cessation of crizotinib. Although the patient was also taking sitagliptin and rabeprazole, crizotinib was the only new medication. The history was notable in this case for chronic inactive hepatitis C infection. A similar case involved a 62-year-old female who presented with markedly elevated serum aminotransferase 17 days after starting crizotinib; therapy was stopped a week later (24 days after initiation), but the patient's clinical status continued to decline and the patient ultimately died of liver failure 40 days after first taking crizotinib [7]. Liver biopsy findings were not presented in these cases.

The histologic findings in this case were notable for a severe lobular hepatitis with submassive parenchymal dropout. The inflammatory infiltrate was not portal-based and the bile ducts themselves were relatively spared. The inflammation was predominantly lymphohistiocytic, but there were no frank granulomata. Neutrophils outnumbered both eosinophils and plasma cells, though the latter two could be identified without much searching. Features to suggest a robust regenerative response—proliferation of residual hepatocytes and widening of the hepatocyte plates—were readily apparent. This finding may have predicted the clinical recovery of liver function seen in this case after the cessation of crizotinib. Paralleling the biochemical evidence of hyperbilirubinemia, there was marked cholestasis that was mostly canalicular and hepatocellular.

This case is also of interest because of the complete resolution of such severe hepatitis, in contrast to the fatal cases described above. Although the exact timing of the onset of liver injury was unknown in the present case, the serum transaminases precipitously declined following the cessation of therapy, suggesting that this intervention was key to the resolution of the injury process. Earlier case reports have shown normalization of more modestly elevated aminotransferases following the withdrawal of crizotinib [8]. A patient with a maximal ALT 1096 IU/L on crizotinib was initially taken off the drug and subsequently underwent successful oral desensitization, tolerating a second course of therapy until the cancer progressed [9].

One salient aspect of this case is the patient-initiated dosing regimen of 500 mg once daily that deviated from the recommended dose during the period preceding the hospitalization for fulminant hepatitis. This regimen reportedly relieved the patient's perceived gastrointestinal discomfort in the setting of reinitiation of crizotinib at 250 mg twice daily following a brief cessation of therapy in the context of pneumonia. In the earliest dose-escalation studies of crizotinib, studied doses ranged from 50 mg once daily to 300 mg twice daily [1]. The effective mean plasma trough concentration of 120 ng per mL, established in preclinical studies, was achieved by dosing regimens of 200 mg or more twice daily. In the cohort receiving 300 mg twice daily, dose-limiting fatigue was noted, leading to the selection of 250 mg twice daily as the maximum tolerated dose. To our knowledge, crizotinib dose-dependent hepatotoxicity has not been observed. It is possible that the 500 mg once daily dosing regimen contributed to the remarkable hepatotoxicity in this case, as well as the favorable clinical response to discontinuation of the drug.

Alternative differential diagnostic considerations in this case included drug/toxin-induced liver injury secondary to another offending agent, although eosinophils did not feature prominently in the inflammatory infiltrate. Furthermore, the patient did not endorse recent exposure to drugs or potential hepatotoxins, aside from crizotinib. Autoimmune hepatitis was also a consideration; the negative anti-smooth muscle, anti-liver-kidney-microsomal, and anti-nuclear antibody studies, and the overall lack of a plasma cell-rich infiltrate, provided no support for an autoimmune etiology. We did not see histologic features to suggest hepatotropic or nonhepatotropic viral infection; also, correlation with viral PCR and serologic studies gave no support for a viral etiology. Notably, the hepatitis resolved without immunosuppression; acyclovir was only briefly given in the face of a positive anti-HSV IgM titer. Taken together, the constellation of findings points to drug-induced liver injury secondary to crizotinib.

This case highlights the potential for complete clinical and biochemical resolution of a fulminant hepatitis arising in association with crizotinib therapy for non-small-cell lung cancer when managed with abrupt cessation of the drug in combination with supportive care, including N-acetylcysteine. Patients should be monitored closely on all chemotherapeutic regimens, such as crizotinib, with prompt evaluation if significant elevations of liver tests are noted; querying whether doses of chemotherapeutic regimens were altered by patients or the treating team may be particularly relevant.
